# Delineating and validating higher-order dimensions of psychopathology in the Adolescent Brain Cognitive Development (ABCD) study

**DOI:** 10.1038/s41398-019-0593-4

**Published:** 2019-10-17

**Authors:** Giorgia Michelini, Deanna M. Barch, Yuan Tian, David Watson, Daniel N. Klein, Roman Kotov

**Affiliations:** 1 Department of Psychiatry & Behavioral Health, Stony Brook University, Stony Brook, NY USA; 2 Departments of Psychological & Brain Sciences, Psychiatry and Radiology, Washington University, St. Louis, MO USA; 3 Department of Applied Mathematics and Statistics, Stony Brook University, Stony Brook, NY USA; 4 Department of Psychology, University of Notre Dame, Notre Dame, IN USA; 5 Department of Psychology, Stony Brook University, Stony Brook, NY USA

**Keywords:** Diseases, Psychiatric disorders

## Abstract

Hierarchical dimensional systems of psychopathology promise more informative descriptions for understanding risk and predicting outcome than traditional diagnostic systems, but it is unclear how many major dimensions they should include. We delineated the hierarchy of childhood and adult psychopathology and validated it against clinically relevant measures. Participants were 9987 9- and 10-year-old children and their parents from the Adolescent Brain Cognitive Development (ABCD) study. Factor analyses of items from the Child Behavior Checklist and Adult Self-Report were run to delineate hierarchies of dimensions. We examined the familial aggregation of the psychopathology dimensions, and the ability of different factor solutions to account for risk factors, real-world functioning, cognitive functioning, and physical and mental health service utilization. A hierarchical structure with a general psychopathology (‘p’) factor at the apex and five specific factors (internalizing, somatoform, detachment, neurodevelopmental, and externalizing) emerged in children. Five similar dimensions emerged also in the parents. Child and parent p-factors correlated highly (*r* = 0.61, *p* < 0.001), and smaller but significant correlations emerged for convergent dimensions between parents and children after controlling for p-factors (*r* = 0.09−0.21, *p* < 0.001). A model with child p-factor alone explained mental health service utilization (*R*^2^ = 0.23, *p* < 0.001), but up to five dimensions provided incremental validity to account for developmental risk and current functioning in children (*R*^2^ = 0.03−0.19, *p* < 0.001). In this first investigation comprehensively mapping the psychopathology hierarchy in children and adults, we delineated a hierarchy of higher-order dimensions associated with a range of clinically relevant validators. These findings hold important implications for psychiatric nosology and future research in this sample.

## Introduction

Traditional psychiatric nosologies define mental disorders as distinct categories^1,2^, but this is at odds with extensive evidence that disorders lie on a continuum with normality and are highly comorbid^3–7^. This comorbidity reflects underlying higher-order dimensions (or spectra) of psychopathology^4,7–9^. Dimensional classifications of these spectra have been proposed as alternative approaches to better align the nosology with empirical evidence^4,7,8,10^. However, available models differ in the number of spectra that they specify.

Numerous studies point to a general factor (‘p’) that represents common susceptibility to psychopathology and explains why all mental disorders tend to co-occur^5,9,11–14^. Other research supports a separation between broad internalizing and externalizing spectra—originally identified in studies that shaped the Achenbach System of Empirically Based Assessment (ASEBA)^15,16^—arguing that this is an important distinction both in adults^17,18^ and children^19,20^. However, further evidence suggests that a greater number of major dimensions are needed to characterize psychopathology^8,21–25^. For instance, the recently developed Hierarchical Taxonomy of Psychopathology (HiTOP)^7,8^ includes six spectra (internalizing, somatoform, detachment, thought disorder, antagonism, and disinhibition), which were identified based on extensive factor analytic literature (for a review, see ref. ^8^). Yet, the dimensions depicted in these studies may not provide full coverage of psychopathology, especially with regard to disorders common in children. For example, a neurodevelopmental spectrum—encompassing forms of psychopathology that share common genetic vulnerabilities, are associated with salient cognitive impairments, emerge in infancy or childhood, and often persist into adulthood (e.g., speech problems, motor problems, autism)—has been proposed^26^, but its placement among other psychopathology spectra remains unclear due to paucity of relevant factor analytic studies^27,28^. Furthermore, the original notion of neurodevelopmental spectrum^26^ did not include problems related to inattention and hyperactivity–impulsivity, which many previous factor analytic studies placed under the externalizing spectrum^29–31^. However, other factor analytic evidence suggests that inattention and hyperactivity–impulsivity symptoms may not cluster with externalizing problems^27,28,32–36^, and accumulating validity studies indicate substantial commonality with other neurodevelopmental problems^37–42^. Further research examining the structure of these symptoms alongside other forms of psychopathology is therefore warranted.

Models with different numbers of dimensions remain to be reconciled in order to advance psychiatric classification and its clinical utility. Simpler and more complex architectures may be integrated as different levels of a single hierarchy: from a p-factor at the apex to progressively more specific nested factors^43,44^. Consequently, models with different numbers of dimensions (one, two, three, etc.) can co-exist and be studied simultaneously. Initial studies, employing Goldberg’s bass-ackwards approach^43^ to delineate hierarchical structures, have identified a hierarchy of higher-order dimensions^22,23,44–46^, but were largely limited to personality pathology and focused on adults. Importantly, developmental studies suggest that some psychopathology dimensions may differ with age, and additional dimensions may emerge over development^14,47^, which underscores the importance of studying child samples as well.

Beyond the identification of the number of dimensions, an important step for delineating a new psychopathology classification is to validate dimensions against criteria important for clinical practice and research, such as genetic/familial and psychosocial risk factors, cognitive processes, illness course, and treatment outcome^48–50^. In a hierarchical structure, validity may differ across levels, as more elaborate models tend to be more informative, but are less parsimonious, and the choice between models may depend on the purpose of inquiry. Available studies show that broader spectra are associated with familiality for psychiatric disorders, childhood adversities, brain and functional impairment^11,13,49^, while more specific dimensions are required to adequately account for outcomes such as educational achievement and executive functioning^14,19,27^. However, a systematic evaluation of validity of dimensions across hierarchical levels is lacking.

In the present study, we sought to delineate higher-order dimensions of psychopathology within a hierarchical structure, and compare the validity of different levels of specificity. Our first aim was to investigate the hierarchical structure of psychopathology in 9987 children from the Adolescent Brain Cognitive Development (ABCD) study^51–53^—as well as in their parents—by analyzing a large and diverse set of symptoms^15,16^. Our second aim was to compare the validity of different levels of the childhood psychopathology hierarchy in relation to clinically informative measures of familial and developmental risk factors, current social, academic, and cognitive functioning, and service utilization^11,14,50,54,55^.

## Methods

### Sample

The ABCD sample consists of over 11,000 children and their parents who took part in a major collaboration between 21 sites across the US to investigate psychological and neurobiological development from preadolescence to early adulthood. Full details of recruitment can be found elsewhere^51^. Briefly, the primary method for recruiting children aged 9 or 10 at the time of the baseline assessments (between 2016 and 2018) and their parents was probability sampling of public and private elementary schools within the catchment areas of the 21 research sites, encompassing over 20% of the entire US population of 9–10 year olds. School selection was based on gender, race and ethnicity, socioeconomic status, and urbanicity. Inclusion criteria were age and attending a public or private elementary school in the catchment area. Exclusion criteria for children were limited to not being fluent in English, having a parent not fluent in English or Spanish, major medical or neurological conditions, gestational age <28 weeks or birthweight <1200 g, contraindications to MRI scanning, a history of traumatic brain injury, a current diagnosis of schizophrenia, moderate/severe autism spectrum disorder, intellectual disability, or alcohol/substance use disorder^56,57^. The cohort’s representation of diverse demographic and socio-economic groups was monitored through the National Center for Education Statistics databases, containing socio-demographic characteristics of the students attending each school, to enable dynamic adjustment of the accumulating sample based on demographic targets throughout recruitment. The final sample who completed the baseline assessment approached the diversity of the US population on several socio-demographic characteristics, despite not being nationally representative^58^: 51% of families were White, 21.4% were Hispanic, 15.2% were African American, 2.3% were Asian, and 10.01% were multiracial or from other ethnical backgrounds; household income was <$50,000 for 30.5% of families, between $50,000 and <$100,000 for 28.1% of families, and at least $100,000 for 41.3% of families; 58.9% of children had at least one parent with a bachelor’s or postgraduate degree; 73.3% parents were married or living in the same household. No weights were applied in the current study. The sample also includes twins recruited from four sites as well as a number of siblings from the same family. However, the present study is based on 9987 unrelated children (randomly selecting one child per family when more than one participated; mean age = 9.90, SD = 0.62; 47.74% females) and 9987 parents (one per child; mean age = 39.94, SD = 6.93; 89.03% females) from the Baseline ABCD 2.0 data release (NDAR-10.15154/1503209). All procedures were approved by a central Institutional Review Board (IRB) at the University of California, San Diego, and in some cases by individual site IRBs (e.g. Washington University in St. Louis)^59^. Parents or guardians provided written informed consent after the procedures had been fully explained and children assented before participation in the study^60^.

### Measures

Full details on measures are presented in Supplementary Method 1. Children and parents completed assessments during an in-person visit. Psychopathology was examined in the children with the parent-reported Child Behavior Checklist (CBCL)^15^ and in the adults with the Adult Self-Report (ASR)^16^ from ASEBA, which assess problems occurring in the past 6 months on a 3-point scale.

For validation, we aimed to select a limited number of validators among those available in the ABCD dataset, based on the two criteria: (1) measures on key, clinically relevant domains, which have commonly been used for validation purposes in previous studies of the structure of psychopathology^11,13,18,55,61^: risk factors, real-world functioning, cognitive functioning, and service utilization; (2) measures that were maximally comprehensive and non-overlapping with each other. Validation analyses therefore focused on the following ten measures: history of developmental motor and speech delays^52^, conflict within the family^62^, social (number of friends) and academic functioning (school connectedness, average grades)^63^, crystalized and fluid intelligence composites from the National Institute of Health Toolbox^53^, utilization of physical and mental health services, and medication use^52^.

### Statistical analysis

To investigate the hierarchical structure of psychopathology, we employed an exploratory approach, given uncertainties regarding the number of dimensions and the composition of the levels of the hierarchy. Specifically, we used exploratory factor analysis (EFA) to empirically extract (with principal component analysis) and rotate (with geomin) factor solutions with an increasing number of factors. We favored an exploratory approach over a confirmatory factor analytic approach as we did not have a-priori hypotheses about the number of factors that would emerge from these data, nor on the exact loading of each item on the factors. To avoid distorting the factor structure in EFA with items that were not analyzable due to being endorsed too infrequently or too-highly correlated with other items, we removed items for which frequency was too low (>99.5% rated 0) and aggregated items that were highly correlated (polychoric *r* > 0.75) into composites (see Supplementary Method 1). The maximum number of factors to extract was determined with parallel analyses^64^ (extraction was stopped when eigenvalues fell within the 95% confidence interval of eigenvalues from simulated data; Supplementary Fig. 1). Since parallel analysis has a tendency to over-factor, we also examined the interpretability of factor solutions^65,66^, defined as presence of >3 clear primary loadings (highest loading ≥0.35 and at least 0.10 greater than all other loadings) for each factor^65,66^. All factor structures from one to the maximum number of factors were considered. To map the hierarchical structure, we correlated factor scores on adjacent levels of the hierarchy to describe transitions between levels using Goldberg’s bass-ackwards hierarchical method^43^. The paths between levels in the hierarchical model reflect correlations ≥0.65 between the factor scores. The bass-ackwards approach was chosen to be consistent with previous studies that investigated the hierarchical structure of psychopathology and personality^22,23,44–46,67,68^, and because, to our knowledge, it is the only method that allows for the delineation of multiple hierarchical levels from factors derived through EFA. Unlike alternative approaches based on bifactor models for extracting a general psychopathology factor (or p-factor) alongside residual specific factors^11,34,36,55^, the bass-ackwards method enables the investigation of multiple levels of a hierarchical structure and the interpretation of factors as interconnected across hierarchical levels, without statistically removing the shared effects of a general factor. In order to take sex into consideration, we further compared factor scores from each hierarchical level in females and males separately in both the child and parent sample.

To compare the utility of the factor solutions, in validation analyses, we first examined the degree of familial aggregation of the dimensions by correlating the factor scores derived for each dimension in parents and children, using both zero-order correlations and partial correlations controlling for the first general psychopathology factors in both parents and children. To examine the familial aggregation due to shared genetic and environmental factors between parent and child, 473 non-biological parent–child pairs were excluded from this analysis (245 adoptive parents, 99 custodial parents, 129 other non-biological parents). Second, we entered the factor scores from each level of the childhood hierarchy as separate blocks into a hierarchical regression model, with each of the validators as the dependent variable. We examined the predictive power and the incremental validity of each level of the hierarchy over more parsimonious structures with the significance of *R*^2^ change between blocks^67^. We used this stringent test, rather than comparing levels in pairs, to ensure that a significant result for models with more factors reflects new information not captured by simpler factor solutions. All analyses were run in Mplus version 7 (Muthén and Muthén, Los Angeles, CA) and SPSS version 25 (IBM Corp, Armonk, NY).

## Results

### Hierarchical factor structure of CBCL and ASR

#### CBCL

Parallel analyses indicated that up to 16 factors could be extracted from CBCL items (Supplementary Fig. 1). After examining the interpretability of these factor solutions, 1- to 5-factor solutions were found to be acceptable (Table 1, Supplementary Table 1). Solutions with more than five factors were not tenable as each included at least one factor with only three or fewer primary loadings (Supplementary Table 1).

**Table 1:** Factor loadings (top) and factor correlations (bottom) for the 5-factor solution from the exploratory factor analysis of CBCL items

	F1	F2	F3	F4	F5
*Primary loading items*					
Composite (Attacks/threatens)	**0.90**	0.03	−0.14	−0.03	0.02
Cruelty, bullying, or meanness to others	**0.88**	−0.05	−0.10	−0.01	−0.03
Composite (Disobeys rules)	**0.81**	−0.11	0.16	−0.01	−0.07
Gets in many fights	**0.78**	−0.13	0.02	0.01	0.05
Temper tantrums or hot temper	**0.77**	0.25	−0.08	0.01	−0.11
Argues a lot	**0.76**	0.18	0.02	0.01	−0.19
Composite (Destroys)	**0.72**	−0.06	0.15	−0.01	0.06
Screams a lot	**0.72**	0.17	−0.03	0.01	−0.04
Doesn’t seem to feel guilty after misbehaving	**0.71**	−0.11	0.11	−0.03	0.05
Swearing or obscene language	**0.70**	−0.01	−0.04	0.02	0.02
Teases a lot	**0.69**	−0.03	0.08	0.06	−0.12
Composite (Steals)	**0.69**	−0.23	0.12	0.01	0.10
Stubborn, sullen, or irritable	**0.69**	0.27	−0.09	0.08	−0.05
Lying or cheating	**0.68**	−0.18	0.16	0.05	0.00
Cruel to animals	**0.67**	−0.05	−0.03	−0.10	0.15
Runs away from home	**0.60**	0.11	0.05	0.00	0.10
Sudden changes in mood or feelings	**0.60**	0.32	−0.02	0.08	0.03
Easily jealous	**0.57**	0.29	0.02	−0.02	−0.07
Composite (Peer problems)	**0.53**	0.07	0.14	−0.03	0.26
Suspicious	**0.53**	0.18	0.08	0.01	0.14
Demands a lot of attention	**0.51**	0.27	0.29	0.00	−0.25
Thinks about sex too much	**0.51**	−0.08	0.12	0.11	0.03
Hangs around with others who get in trouble	**0.51**	−0.18	0.19	0.04	0.01
Feels others are out to get him/her	**0.50**	0.36	0.00	−0.05	0.11
Sets fires	**0.50**	−0.18	0.19	−0.08	0.06
Sulks a lot	**0.49**	0.34	−0.09	0.12	0.12
Showing off or clowning	**0.49**	−0.04	0.36	0.04	−0.28
Bragging, boasting	**0.48**	0.05	0.21	0.08	−0.31
Whining	**0.41**	0.27	0.09	0.09	−0.09
Too fearful or anxious	−0.13	**0.70**	0.33	0.02	0.03
Worries	−0.06	**0.67**	0.18	0.13	0.00
Feels he/she has to be perfect	−0.01	**0.67**	−0.01	0.00	−0.02
Feels too guilty	−0.02	**0.65**	0.18	0.06	−0.01
Nervous, high-strung, or tense	0.04	**0.57**	0.38	0.00	−0.04
Fears he/she might think or do something bad	0.05	**0.56**	0.19	−0.03	0.04
Feels worthless or inferior	0.28	**0.55**	0.04	−0.03	0.14
Self-conscious or easily embarrassed	0.06	**0.46**	0.07	0.08	0.26
Fears going to school	0.09	**0.40**	0.08	0.10	0.27
Fears certain animals, situations, or places, other than school	−0.05	**0.37**	0.24	0.08	0.08
Complains of loneliness	0.25	**0.36**	0.16	0.04	0.13
Composite (Distracted/Hyperactive)	0.21	−0.04	**0.77**	−0.06	0.00
Daydreams or gets lost in his/her thoughts	−0.12	0.05	**0.64**	0.02	0.20
Stares blankly	−0.03	−0.03	**0.60**	0.04	0.36
Confused or seems to be in a fog	−0.06	0.07	**0.60**	−0.02	0.36
Poorly coordinated or clumsy	−0.02	−0.08	**0.58**	0.24	0.15
Nervous movements or twitching	−0.01	0.24	**0.54**	0.00	−0.02
Fails to finish things he/she starts	0.27	−0.01	**0.53**	0.02	0.05
Talks too much	0.20	0.04	**0.52**	0.12	−0.26
Can’t get his/her mind off certain thoughts; obsessions	0.16	0.30	**0.50**	−0.05	−0.02
Poor school work	0.29	−0.14	**0.49**	−0.04	0.18
Repeats certain acts over and over; compulsions	0.20	0.11	**0.48**	−0.03	0.14
Strange ideas	0.18	0.04	**0.45**	0.04	0.18
Acts too young for his/her age	0.20	0.05	**0.45**	−0.09	0.12
Gets hurt a lot, accident prone	0.04	−0.06	**0.41**	0.31	−0.02
Prefers being with younger kids	0.11	0.05	**0.35**	0.03	0.19
Nausea, feels sick	−0.01	0.04	−0.06	**0.89**	−0.05
Stomachaches	−0.01	0.05	−0.08	**0.82**	−0.03
Vomiting, throwing up	0.02	−0.19	−0.04	**0.75**	0.06
Headaches	0.02	0.02	−0.02	**0.62**	0.01
Aches or pains (not stomach or headaches)	0.00	0.05	0.05	**0.57**	−0.01
Feels dizzy or lightheaded	−0.05	0.15	0.08	**0.53**	0.11
Other (physical problems without known physical cause)	0.02	0.04	0.11	**0.48**	0.02
Problems with eyes (not if corrected by glasses)	0.00	−0.04	0.04	**0.36**	0.23
Rashes or other skin problems	0.01	0.00	0.10	**0.35**	0.04
Withdrawn, doesn’t get involved with others	0.17	0.17	0.03	0.05	**0.65**
Would rather be alone than with others	0.13	0.11	0.05	0.01	**0.56**
Too shy or timid	−0.10	0.31	−0.01	0.06	**0.55**
Refuses to talk	0.27	0.13	−0.02	0.04	**0.51**
Underactive, slow moving, or lacks energy	0.07	0.00	0.12	0.34	**0.45**
*Non-primary loading or cross-loading items*					
Secretive, keeps things to self	0.32	0.08	0.02	0.08	0.40
Strange behavior	0.32	0.05	0.41	−0.01	0.23
There is very little he/she enjoys	0.39	0.16	0.01	0.01	0.34
Unhappy, sad, or depressed	0.38	0.42	−0.09	0.12	0.23
Unusually loud	0.39	0.08	0.42	0.11	−0.21
Deliberately harms self or attempts suicide	0.39	0.37	0.06	−0.08	0.10
Feels or complains that no one loves him/her	0.54	0.47	−0.10	−0.05	0.07
Impulsive or acts without thinking	0.49	0.02	0.49	−0.05	−0.11
Talks about killing self	0.44	0.38	−0.01	−0.03	0.05
Overtired without good reason	0.14	0.07	0.07	0.35	0.32
Composite (Sex play)	0.33	−0.03	0.17	0.03	−0.01
Composite (Weight problems)	0.14	−0.01	0.04	0.22	0.16
Composite (Hallucinations)	0.16	0.01	0.26	0.20	0.18
Bowel movements outside toilet	0.13	−0.07	0.12	0.14	0.19
Trouble sleeping	0.04	0.25	0.30	0.23	0.01
Wets self during the day	0.08	−0.01	0.25	0.12	0.17
Wets the bed	0.13	−0.10	0.15	0.07	0.06
Wishes to be of opposite sex	0.07	0.11	0.11	−0.01	0.24
Clings to adults or too dependent	0.13	0.28	0.29	0.06	0.10
Cries a lot	0.31	0.31	0.10	0.05	0.08
Doesn’t eat well	0.16	0.08	0.16	0.12	0.10
Gets teased a lot	0.30	0.06	0.23	0.04	0.28
Bites fingernails	0.07	0.10	0.23	0.05	−0.04
Nightmares	0.03	0.19	0.27	0.27	−0.03
Constipated, doesn’t move bowels	−0.01	0.13	0.11	0.29	0.09
Picks nose, skin, or other parts of body	0.17	0.09	0.33	0.08	−0.04
Prefers being with older kids	0.30	−0.02	0.20	0.11	0.03
Sleeps less than most kids	0.06	0.16	0.33	0.15	0.04
Sleeps more than most kids during day and/or night	0.10	−0.01	0.11	0.21	0.26
Speech problem	0.00	−0.07	0.34	−0.01	0.23
Stores up too many things he/she doesn’t need	0.18	0.13	0.25	0.11	0.04
Talks or walks in sleep	0.02	0.03	0.24	0.25	−0.14
Thumb-sucking	0.11	−0.03	0.07	0.07	0.01
*Factor correlations*					
F1 (Externalizing)	1				
F2 (Internalizing)	0.33	1			
F3 (Neurodevelopmental)	0.59	0.33	1		
F4 (Somatoform)	0.38	0.44	0.38	1	
F5 (Detachment)	0.35	0.34	0.36	0.25	1

Bold indicates primary loadings (≥0.35) with at least 0.10 difference from the second largest loading. All factor correlations were statistically significant (*p* < 0.001, two-tailed)

*CBCL* Child Behavior Checklist, *F1* externalizing factor, *F2* internalizing factor, *F3* neurodevelopmental factor, *F4* somatoform factor, *F5* detachment factor

**Fig. 1:**
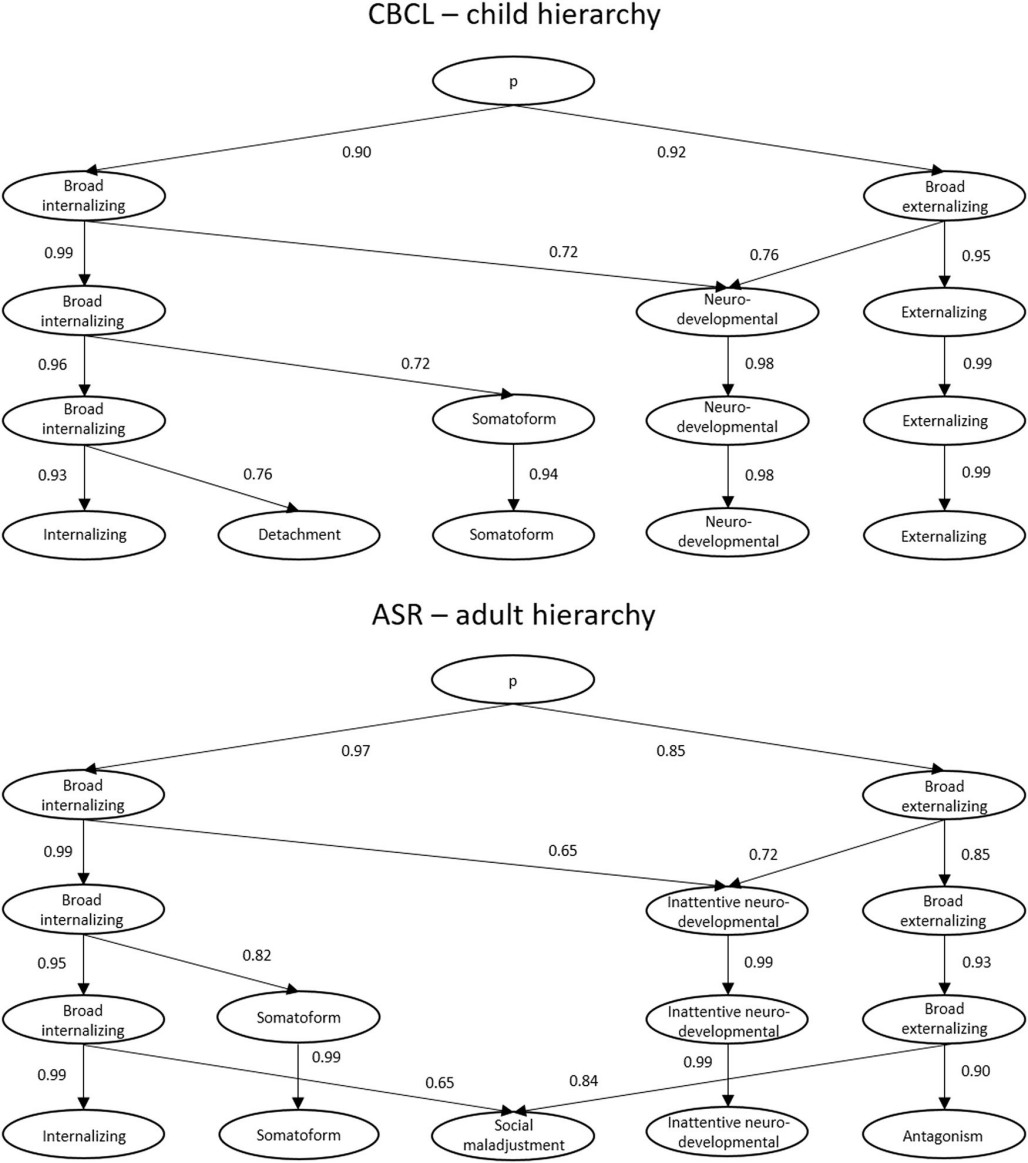
Hierarchical models from CBCL items (top half) and ASR items (bottom half) illustrating hierarchies of child and adult psychopathology. CBCL Childhood Behavior Checklist, ASR Adult Self Report

All models from 1-factor to 5-factor were interpretable and are represented as a hierarchical structure (Fig. 1), with paths showing correlations between levels. The 1-factor structure reflected a general childhood psychopathology p-factor^5,14^. The 2-factor solution revealed the expected broad internalizing and broad externalizing factors^15,19,68^. In the 3-factor structure, a neurodevelopmental factor (e.g. inattention, hyperactivity, daydreaming, clumsiness) emerged from the broad internalizing and externalizing factors. In the 4-factor solution, somatoform problems emerged from the broad internalizing factor. In the 5-factor structure, the remaining broad internalizing factor split into narrower internalizing problems (e.g. anxiety, depressive symptoms) and detachment (e.g. social withdrawal). Factors in the final 5-factor solution showed small-to-large correlations with one another (*r* = 0.25−0.59) (Table 1). Comparisons of factor scores across boys and girls indicated small but highly significant (all *p* ≤ 0.001) sex differences on all dimensions, except broad internalizing in the 2-, 3-, and 4-factor solutions (Supplementary Table 2). Boys showed slightly higher psychopathology on the p-factor, as well as externalizing, neurodevelopmental, and detachment factors in the 5-factor solution, while girls had slightly higher scores on internalizing and somatoform factors.

#### ASR

Parallel analyses indicated that up to 17 factors could be extracted from ASR items (Supplementary Fig. 1). The 5-factor solution was the most differentiated interpretable structure (Table 2, Supplementary Table 3), as factor solutions with more factors could not be interpreted. For example, the last factor in the 6- and 8-factor models included only two-to-three primary loadings, thus indicating no other meaningful factors beyond five (Supplementary Table 3).

**Table 2:** Factor loading (top) and factor correlations (bottom) for the 5-factor solution from the exploratory factor analysis on ASR items

	F1	F2	F3	F4	F5
*Primary loading items*					
I lack self-confidence	**0.73**	0.05	0.22	−0.07	−0.13
I worry a lot	**0.72**	−0.11	−0.04	0.15	0.18
I am self-conscious or easily embarrassed	**0.72**	0.06	0.06	−0.05	−0.08
I feel worthless and inferior	**0.69**	0.22	0.11	−0.03	−0.04
Composite (Anxious)	**0.67**	−0.12	0.12	0.13	0.13
I feel too guilty	**0.65**	−0.09	0.15	0.03	0.13
I feel that I have to be perfect	**0.62**	−0.10	−0.04	−0.09	0.16
I am jealous of others	**0.58**	−0.04	0.19	−0.24	0.18
I am too shy or timid	**0.55**	0.26	−0.01	−0.03	−0.27
I am unhappy, sad, or depressed	**0.54**	0.22	0.06	0.20	−0.02
I feel overwhelmed by my responsibilities	**0.54**	−0.13	0.25	0.07	0.13
I worry about my family	**0.53**	−0.06	−0.08	0.15	0.17
I feel lonely	**0.52**	0.30	0.04	0.05	−0.03
I worry about my future	**0.52**	0.06	−0.02	0.06	0.09
I feel that I can’t succeed	**0.46**	0.20	0.22	0.01	−0.07
I can’t get my mind off certain thoughts	**0.40**	0.06	0.14	0.15	0.19
I blame others for my problems	**0.39**	0.10	0.18	−0.24	0.24
I cry a lot	**0.39**	0.20	−0.03	0.18	0.12
I refuse to talk	0.21	**0.60**	−0.01	0.09	−0.04
I have trouble making or keeping friends	0.38	**0.58**	0.05	−0.08	−0.12
I do things that may cause me trouble with the law	−0.15	**0.57**	0.24	−0.05	0.20
I am not liked by others	0.28	**0.57**	0.00	−0.05	0.06
I don’t get along with other people	0.15	**0.56**	−0.06	−0.01	0.15
My relations with the opposite sex are poor	0.25	**0.54**	−0.01	0.01	−0.02
I am secretive or keep things to myself	0.20	**0.54**	−0.07	0.11	−0.02
I keep from getting involved with others	0.25	**0.53**	−0.02	0.03	−0.13
Composite (Hallucinations)	−0.03	**0.52**	−0.05	0.34	0.09
I steal	−0.07	**0.51**	0.23	−0.13	0.14
Composite (Vandalism)	0.02	**0.49**	0.00	0.07	0.29
I have trouble keeping a job	0.04	**0.48**	0.22	0.05	0.03
My relations with neighbors are poor	0.11	**0.48**	0.02	0.06	0.00
I hang around people who get into trouble	−0.13	**0.47**	0.19	0.08	0.20
I feel that others are out to get me	0.32	**0.46**	−0.06	0.09	0.17
I lie or cheat	0.02	**0.44**	0.20	−0.10	0.19
Composite (Oddness)	−0.01	**0.43**	0.19	0.10	0.15
I would rather be with older people than with people of my own age	0.04	**0.43**	−0.04	0.22	0.04
I think about sex too much	−0.06	**0.43**	0.13	−0.02	0.21
I would rather be alone than with others	0.30	**0.42**	−0.02	0.06	−0.12
I get along badly with my family	0.26	**0.42**	−0.04	0.00	0.15
I wish I were of the opposite sex	0.08	**0.41**	0.11	0.14	0.01
I break rules at work or elsewhere	−0.08	**0.39**	0.29	−0.12	0.24
I use drugs (other than alcohol, nicotine) for nonmedical purposes	−0.12	**0.39**	0.14	−0.06	0.10
I repeat certain acts over and over	0.10	**0.38**	0.07	0.18	0.16
I don’t feel guilty after doing something I shouldn’t	−0.10	**0.38**	0.06	−0.02	0.14
I have a speech problem	−0.03	**0.36**	0.19	0.16	−0.07
People think I am disorganized	−0.01	0.00	**0.75**	0.07	0.01
I have trouble setting priorities	0.18	0.06	**0.72**	−0.04	−0.05
I fail to finish things I should do	0.19	0.05	**0.68**	0.01	−0.05
I tend to lose things	0.03	−0.03	**0.59**	0.22	0.06
I am not good at details	0.03	0.09	**0.56**	−0.01	0.00
I have trouble concentrating or paying attention for long	0.09	−0.06	**0.56**	0.21	0.09
I am too forgetful	0.06	−0.07	**0.54**	0.24	0.01
My work performance is poor	0.21	0.26	**0.49**	0.04	−0.10
I tend to be late for appointments	0.01	−0.04	**0.49**	0.02	0.08
I have trouble planning for the future	0.29	0.20	**0.46**	0.07	−0.07
I rush into things without considering the risks	−0.05	0.23	**0.40**	0.04	0.27
Composite (Money management)	0.09	0.22	**0.36**	0.11	0.05
Composite (Nausea)	−0.02	0.11	0.02	**0.73**	0.00
Stomachaches	0.02	0.03	0.00	**0.69**	0.03
Aches or pains (not stomach or headaches)	−0.06	0.05	0.08	**0.67**	0.03
Headaches	0.08	−0.06	−0.05	**0.64**	0.02
Numbness or tingling in body parts	0.00	0.08	0.05	**0.62**	0.04
I feel dizzy or lightheaded	0.14	0.04	0.06	**0.55**	0.01
Heart pounding or racing	0.20	0.04	0.03	**0.52**	0.04
Problems with eyes (not if corrected by glasses)	−0.09	0.20	−0.02	**0.48**	0.01
I feel tired without good reason	0.26	0.04	0.25	**0.45**	−0.08
I don’t have much energy	0.29	0.00	0.27	**0.43**	−0.11
Rashes or other skin problems	0.02	0.04	0.07	**0.38**	0.02
I have trouble sleeping	0.23	0.04	0.05	**0.37**	0.07
Parts of my body twitch or make nervous movements	0.12	0.17	0.16	**0.37**	0.09
I am louder than others	−0.05	−0.09	0.22	0.05	**0.63**
I have a hot temper	0.29	0.12	−0.07	0.02	**0.61**
I talk too much	−0.01	−0.22	0.29	0.08	**0.56**
I argue a lot	0.31	0.06	−0.03	−0.09	**0.55**
I scream or yell a lot	0.31	0.08	−0.07	0.04	**0.54**
I am too impatient	0.32	−0.03	0.17	0.00	**0.49**
I tease others a lot	0.00	0.05	0.25	−0.12	**0.47**
I try to get a lot of attention	0.02	0.10	0.26	−0.16	**0.45**
I show off or clown	−0.17	0.12	0.26	−0.06	**0.42**
I brag	−0.03	0.14	0.17	−0.13	**0.40**
*Non-primary loading or cross-loading items*					
I have trouble sitting still	0.03	−0.03	0.32	0.17	0.30
I feel restless or fidgety	0.19	0.03	0.28	0.30	0.25
I dislike staying in one place for very long	−0.03	0.23	0.11	0.16	0.19
I feel that no one loves me	0.53	0.47	−0.11	0.03	0.00
There is very little I enjoy	0.37	0.44	0.08	0.13	−0.09
Composite (Suicidality)	0.37	0.42	0.06	0.06	−0.03
I get in many fights	0.08	0.37	−0.01	0.07	0.43
I am mean to others	0.09	0.37	−0.03	−0.07	0.40
I sleep more than most other people during day and/or night	0.08	0.17	0.19	0.30	−0.06
I stay away from my job even when I’m not sick or not on vacation	−0.04	0.35	0.26	0.09	0.02
I worry about my relations with the opposite sex	0.34	0.35	0.07	0.00	0.05
I get upset too easily	0.51	0.05	−0.01	0.05	0.48
I am too dependent on others	0.36	0.09	0.32	−0.02	0.04
I drive too fast	−0.01	0.04	0.26	−0.03	0.26
I feel confused or in a fog	0.32	0.10	0.31	0.28	0.00
I daydream a lot	0.11	0.15	0.27	0.09	0.05
I don’t eat as well as I should	0.21	0.00	0.21	0.18	0.05
I am afraid of certain animals, situations, or places	0.19	0.20	−0.08	0.20	0.03
I am afraid I might think or do something bad	0.38	0.33	0.06	−0.01	0.09
I am impulsive or act without thinking	0.07	0.22	0.32	0.07	0.34
I pick my skin or other parts of my body	0.18	0.01	0.22	0.05	0.09
My behavior is irresponsible	0.05	0.40	0.39	0.00	0.14
I have trouble making decisions	0.45	−0.05	0.47	−0.03	−0.06
My behavior is very changeable	0.06	0.31	0.10	0.07	0.21
I am easily bored	0.04	0.30	0.12	0.12	0.23
I am stubborn, sullen, or irritable	0.37	0.15	−0.03	0.08	0.38
I drink too much alcohol or get drunk	−0.02	0.17	0.23	−0.09	0.15
Composite (Clumsiness)	0.09	0.03	0.33	0.28	0.06
Composite (Moods wings)	0.31	0.29	0.03	0.24	0.26
Composite (Overt aggression)	0.01	0.45	−0.01	0.17	0.43
*Factor correlations*					
F1 (Internalizing)					
F2 (Social maladjustment)	0.43				
F3 (Inattentive neurodevelopmental)	0.44	0.45			
F4 (Somatoform)	0.50	0.40	0.32		
F5 (Antagonism)	0.19	0.41	0.31	0.27	

Bold indicates primary loadings (≥0.35) with at least 0.10 difference from the second largest loading. All factor correlations were statistically significant (*p* < 0.05, two-tailed)

*ASR* Adult Self Report, *F1* internalizing factor, *F2* social maladjustment factor, *F3* inattentive neurodevelopmental factor, *F4* somatoform factor, *F5* antagonism factor

All models from 1-factor to 5-factor are represented in Fig. 1. The 1-factor structure reflected p-factor^11^. The 2-factor solution showed the broad internalizing and externalizing factors^17^. In the 3-factor structure, a factor encompassing inattentive neurodevelopmental problems (e.g. inattention, poor planning) emerged from the broad internalizing and externalizing factors. In the 4-factor solution, the broad internalizing factor split into separate internalizing and somatoform dimensions. In the 5-factor structure, rule-breaking behaviors from the broad externalizing factor joined detachment/oddity problems from the broad internalizing factor to form a social maladjustment factor, leaving distinct antagonism and narrower internalizing dimensions. Factors in the final 5-factor solution showed small-to-large correlations with one another (*r* = 0.19−0.50) (Table 2). Comparisons of factor scores across women and men indicated small but highly significant (all *p* ≤ 0.001) sex differences on all but the inattentive neurodevelopmental factor in the 3-, 4-, and 5-factor solutions (Supplementary Table 2). Women scored higher than men on the p-factor, as well as on the internalizing, somatoform factors in the 5-factor solution, while men showed higher scores on the social maladjustment and antagonism factors.

### Validation analyses

#### Familial aggregation

Zero-order correlations between the child and adult factor scores from the 5-factor solutions ranged between *r* = 0.20−0.48 (*p* < 0.001, two-tailed) (Table 3). The correlation between child and parent p-factor scores was *r* = 0.61 (*p* < 0.001, two-tailed). This pattern suggested substantial familial aggregation of a dimension of general psychopathology, explaining co-occurrence across psychopathology dimensions. Controlling for these two p-factors revealed a more specific pattern of familial aggregation between corresponding parent and child dimensions (i.e. convergent correlations). Convergent partial correlations ranged between *r* = 0.09−0.21 (*p* < 0.001, two-tailed) and were significantly larger than all partial correlations between non-corresponding factors (i.e. discriminant correlations), based on Fisher’s *z* tests (Table 3).

**Table 3:** Zero-order (top half) and partial correlations (bottom half) between the dimensions in the 5-factor structures from CBCL and ASR items, controlling for childhood and adult p-factors

	ASR				
	Internalizing	Social maladjustment	Inattentive neurodevelopmental	Somatoform	Antagonism
Zero-order correlations					
CBCL					
Externalizing	0.42	**0.44**	0.40	0.38	**0.38**
Internalizing	**0.45**	0.27	0.33	0.33	0.24
Neurodevelopmental	0.42	0.41	**0.43**	0.40	0.34
Somatoform	0.42	0.34	0.37	**0.48**	0.27
Detachment	0.34	**0.38**	0.31	0.31	0.20
Partial correlations					
CBCL					
Externalizing	−0.09	**0.12**	−0.01	−0.06	**0.11** ^b^
Internalizing	**0.19** ^a,b^	−0.17	−0.04	−0.02	−0.07
Neurodevelopmental	−0.09	0.04	**0.09** ^a,b^	0.01	0.01
Somatoform	0.00	−0.09	−0.03	**0.21** ^a,b^	−0.07
Detachment	0.00	**0.13** ^a^	−0.02	0.00	−0.10

Bold denotes convergent correlations between child and parent dimensions. All zero-order correlations are statistically significant (*p* < 0.001, two-tailed). Partial correlations *r* ≥ |0.03| are significant (*p* < 0.05, two-tailed)

*ASR* Adult Self Report, *CBCL* Childhood Behavior Checklist

^a^Indicates a partial correlation that is significantly higher than all others in the row based on Fisher’s *z* tests

^b^Indicates a partial correlation that is significantly higher than all others in the column based on Fisher’s *z* tests

#### Validity of childhood hierarchical structure

**Fig. 2:**
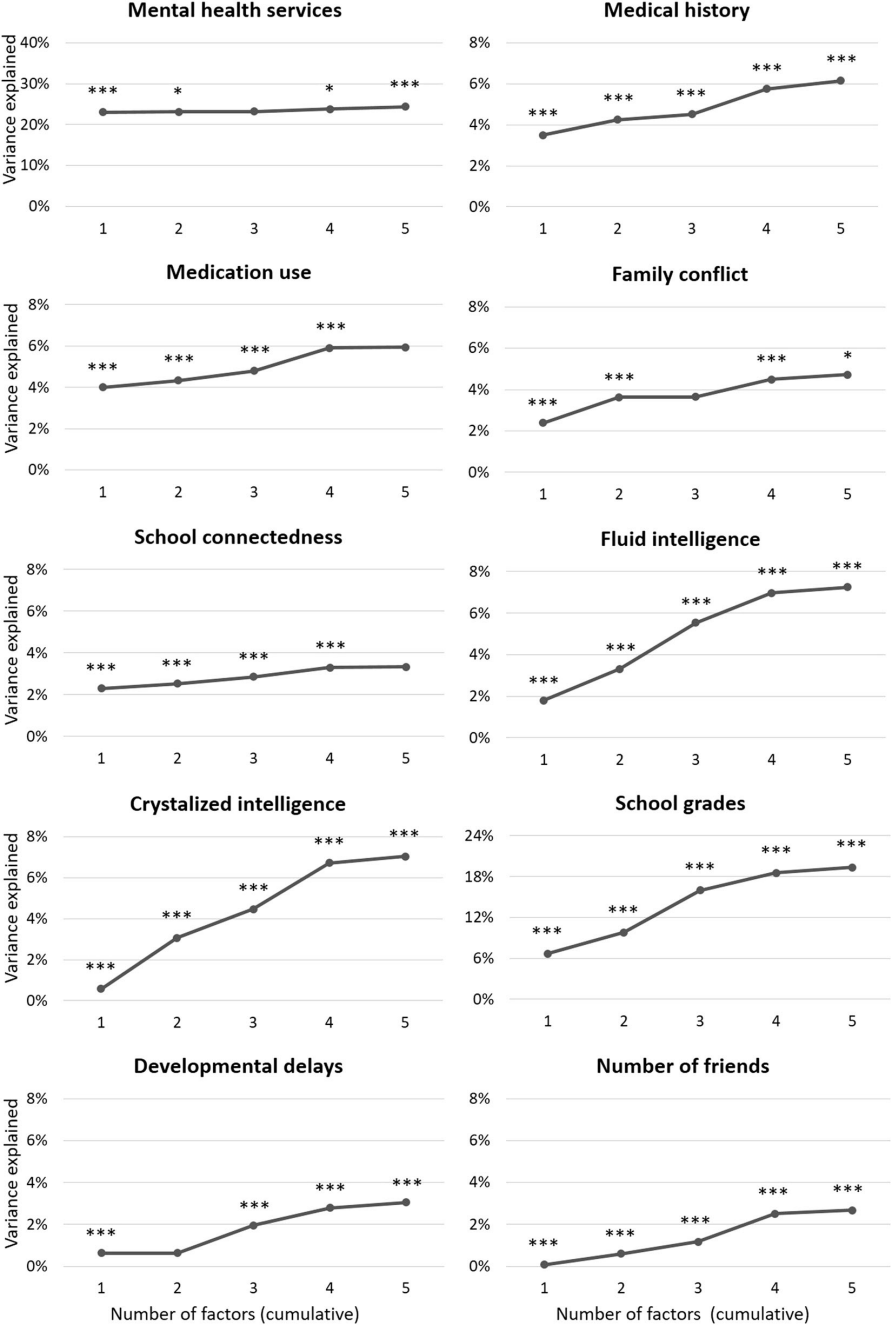
Cumulative explanatory power (*R*^2^) for a given factor structure (1- to 5-factor solutions) derived from CBCL data to predict validators. Nagelkerke *R*^2^ is plotted for binary outcomes (mental health service utilization, medical history, medication history). Asterisks indicate significant change in *R*^2^ for that structure versus all simpler structures combined (*p < 0.05, **p < 0.01, ***p < 0.001, two-tailed). CBCL Childhood Behavior Checklist

The 1-factor solution was significantly associated with all validators (Fig. 2, Supplementary Table 4). The p-factor alone explained 23.02% of the variance in utilization of mental health services, and the addition of more differentiated factors, although statistically significant, produced minimal improvement in *R*^2^ (up to 24.41%). For medication use, medical history, family conflict, and school connectedness, the p-factor alone explained 2.30–4.00% of the variance, and the addition of more complex factor structures provided a moderate increase, contributing up to 3.33–6.16% of variance. The 1-factor model accounted for a relatively small proportion of the variance compared to the more complex factor solutions for fluid intelligence (from 1.79% for p-factor to 7.24% total), crystalized intelligence (0.58% to 7.02%), average grades (6.72% to 19.34%), number of friends (0.08% to 2.67%), and history of developmental delays (0.63% to 3.05%).

In the 5-factor solution, utilization of mental health services showed the highest but generally non-specific correlations with psychopathology dimensions (*r* = 0.28–0.46) (Supplementary Table 4). The strongest association for medical history was with the somatoform factor (*r* = 0.26). Medication use was associated to the same extent with the neurodevelopmental and somatoform factors (both *r* = 0.22). Crystalized intelligence and school connectedness were associated to a similar extent with the externalizing (*r* = −0.12), neurodevelopmental, and detachment factors (both *r* = −0.11). The highest correlation for family conflict was with the externalizing factor (*r* = 0.19). Fluid intelligence and average grades showed the highest correlations with the neurodevelopmental factor (*r* = −0.18 and *r* = −0.33, respectively). Developmental delays were mostly associated with the detachment and neurodevelopmental factors (*r* = 0.14 and *r* = 0.10, respectively), while number of friends were mostly associated with detachment (*r* = −0.13).

## Discussion

This study provides the most comprehensive examination of the hierarchy of psychopathology spectra to date—analyzing a wide range of symptoms and maladaptive behaviors, systematically explicating it across multiple hierarchical levels, considering both children and adults, and validating the structure against various clinically relevant measures. In children, we found five spectra at the lowest level of the hierarchy: internalizing, somatoform, detachment, externalizing, and neurodevelopmental. In adults, we observed similar dimensions: internalizing, somatoform, social maladjustment, inattentive neurodevelopmental, and antagonism. We further found substantial familiality of the identified psychopathology factors, largely explained by familial aggregation of the p-factor. Yet, the five childhood dimensions also showed specific links to the corresponding parental dimensions. The p-factor was sufficient to account for some clinical validators (e.g., service utilization), but all five dimensions were needed to explain other validators, such as developmental delays, and social, cognitive, and academic functioning. These findings support the value of explicating multiple higher-order dimensions of psychopathology. They further suggest that the neurodevelopmental spectrum should be considered for inclusion in dimensional models of both childhood and adult psychopathology. Overall, the identified hierarchy depicts robust and informative dimensional phenotypes for the ABCD study baseline assessment, paving the way for future research on this cohort.

In both children and adults, we observed that the p-factor at the top of the hierarchy separates into broad internalizing and broad externalizing spectra. These dimensions mirror the higher-order dimensions first identified by Achenbach and colleagues^32^. At lower hierarchical levels, the broad internalizing dimension differentiated into internalizing, detachment, and somatoform factors in children. The broad externalizing factor differentiated into narrower externalizing and neurodevelopmental factors in children (the latter originating from both broad externalizing and internalizing). The narrow externalizing factor included aggressive and rule-breaking behaviors, whereas the neurodevelopmental factor encompassed inattention, hyperactivity, and related problems (e.g. clumsiness, daydreaming, obsessions). In adults, instead of detachment as a distinct factor, we found a broader social maladjustment factor encompassing both detachment and antisocial behavior (from the externalizing factor). The differentiation of the externalizing spectrum in adults narrowed to one of its core components, antagonism, which emerged separately from the social maladjustment and inattentive neurodevelopmental factors (both partly originating from the broad internalizing spectrum). This is in line with research showing a separation of antagonism from other externalizing and neurodevelopmental dimensions^8,69,70^. All observed dimensions are consistent with prior studies, which have identified these factors among major dimensions of psychopathology^8,25,32,71^. Overall, similar but not identical dimensions were delineated in children and parents, which does not support the hypothesis that psychopathology becomes more differentiated with age^14,47^.

Our findings are largely consistent with the HiTOP model^7,8,72^, in that internalizing, antagonism, somatoform, and detachment dimensions were identified in children and/or adults. The adult social maladjustment dimension identified here has the HiTOP detachment spectrum at its core, along with additional content relating to antisocial behavior and a few symptoms of thought disorder (e.g. hallucinations). A thought disorder spectrum was not found either in adults or in children, likely because of the limited number of psychosis symptoms included in the CBCL and ASR, the very low scores on these symptoms in this population-based sample, and the exclusion of children with a diagnosis of schizophrenia based on ABCD recruitment procedures. We observed an additional factor in children that is currently not included in the HiTOP model: a neurodevelopmental dimension that includes inattention, hyperactivity, clumsiness, autistic-like traits, and atypical ideation (e.g. obsessions). Many of the symptoms included in this dimension have previously been proposed to be part of a neurodevelopmental spectrum^26^ and are consistent with initial factor analytic evidence in children^27,28,49^. Our results indicate that inattentive and hyperactive symptoms (common in attention-deficit/hyperactivity disorder (ADHD)) also belong to this spectrum, despite previous studies that included ADHD as part of the externalizing spectrum^29,31^. One explanation for this finding is that many previous EFA studies placing ADHD under the externalizing spectrum examined scale total scores or diagnoses, rather than individual symptoms, and did not include other neurodevelopmental problems—thereby not allowing the delineation of a separate dimension. The emergence of a similar, though narrower, inattentive neurodevelopmental factor in adults is novel, as most previous structural studies of adults have not considered enough attention or neurodevelopmental problems to allow the delineation of this dimension. This finding provides the strongest evidence to date for the inclusion of the neurodevelopmental spectrum in dimensional models of psychopathology. More generally, these findings delineating dimensions of psychopathology both in children and in adults in one of the largest samples available to date represent an important contribution to ongoing efforts seeking to understand the hierarchical structure of psychopathology. Future studies on this cohort and other samples may employ alternative analytic approaches (e.g. bifactor models)^11,34,36,55^ and instruments (e.g. diagnostic interviews) to examine the reproducibility of the identified dimensions and further advance knowledge of the structure of psychopathology.

By mapping multiple hierarchical levels, we showed that the familial aggregation of psychopathological dimensions in parents and children is largely accounted for by familial influences on the p-factor. This is consistent with the established pleiotropy in the genetic vulnerability to psychopathology^19,73,74^ and prior evidence of substantial heritability of the p-factor^5^. In children, the p-factor also accounted for the majority of psychopathology-related variance in several validators, especially utilization of mental health services, which underscores the value of this general dimension for public health and planning of clinical services. However, more specific dimensions also proved to be informative. Familial aggregation between specific dimensions remained significant, albeit reduced, when controlling for child and parent p-factors, and all levels of the hierarchy showed incremental validity, with five dimensions necessary to maximize the explanatory power of psychopathology for most criteria. This supports the importance of examining multiple levels of the psychopathology hierarchy, and is consistent with the view that fine-grained understanding of psychopathology is necessary to fully explicate its etiology^75,76^ and identify maximally effective treatment^77^. Further, different dimensions were most important for different validators. For example, the neurodevelopmental dimension had particularly strong links to intelligence and academic achievement, consistent with previous evidence^78,79^, and the externalizing factor with family conflict, as expected^54^. These results confirm previous studies showing that both a general factor and specific dimensions are necessary for characterizing youth psychopathology^19^, school grades, school and neighborhood deprivation^14^, and executive functioning^27^. They are inconsistent with studies linking cognitive abilities primarily to the p-factor^11,55^, potentially because these studies did not model the neurodevelopmental dimension, the strongest correlate of fluid intelligence in this study.

The present study had the following limitations. First, it was limited to one assessment system, thus generalizability of the findings needs to be tested with other measures. Nevertheless, the hierarchy is largely consistent with previous studies using different measures^21,23,44,69^, suggesting at least partial generalizability. Second, the same parent completed both the CBCL about the child and the ASR about themselves, which may have inflated the similarity between childhood and adult psychopathology structures due to rater biases. In addition, most of the ASR data were provided by mothers or female guardians, therefore the results in the adult sample may not generalize to both sexes. Although these limitation are common to much of the existing literature on parent and offspring psychopathology when children are too young to provide comprehensive self-reports, and a number of our validators were objective (e.g. cognitive testing) or child self-report (e.g. number of friends) measures, future research should replicate the current results with child self-reports and additional co-informant reports. Third, only one time point was included, as longitudinal data were not yet available from the ABCD study at the time of writing. Future waves of data in this unique sample will provide the unprecedented opportunity to examine the hierarchy of psychopathology over the course of development and the predictive validity of childhood factors on a variety of adolescent and young adult outcomes.

In conclusion, the present results clarify the hierarchy of psychopathology dimensions in children and adults using data from one of the largest initiatives to study youth development and psychopathology to date. The study replicates higher-order dimensions identified previously^8^, and suggests the addition of the neurodevelopmental spectrum to dimensional models of psychopathology. The identified higher-order dimensions represent valid constructs able to explain various clinically relevant risk factors and outcomes, such as developmental delays and academic achievement. Our investigation further provides a guide for future research to use these higher-order psychopathology dimensions in the ABCD sample. New data releases will allow researchers to apply the identified hierarchy to additional clinical, functional, and neuroimaging measures to study psychopathological dimensions during adolescent development.

## Supplementary information

Supplementary Method 1

Supplementary Figure 1

Supplementary Table 1

Supplementary Table 2

Supplementary Table 3

Supplementary Table 4
