# Supplementary figures and images for: Delineating and validating higher-order dimensions of psychopathology in the Adolescent Brain Cognitive Development (ABCD) study
Source: Transl Psychiatry. 2019 Oct 17;9:261. doi: 10.1038/s41398-019-0593-4 (PMC6797772; doi:10.1038/s41398-019-0593-4)

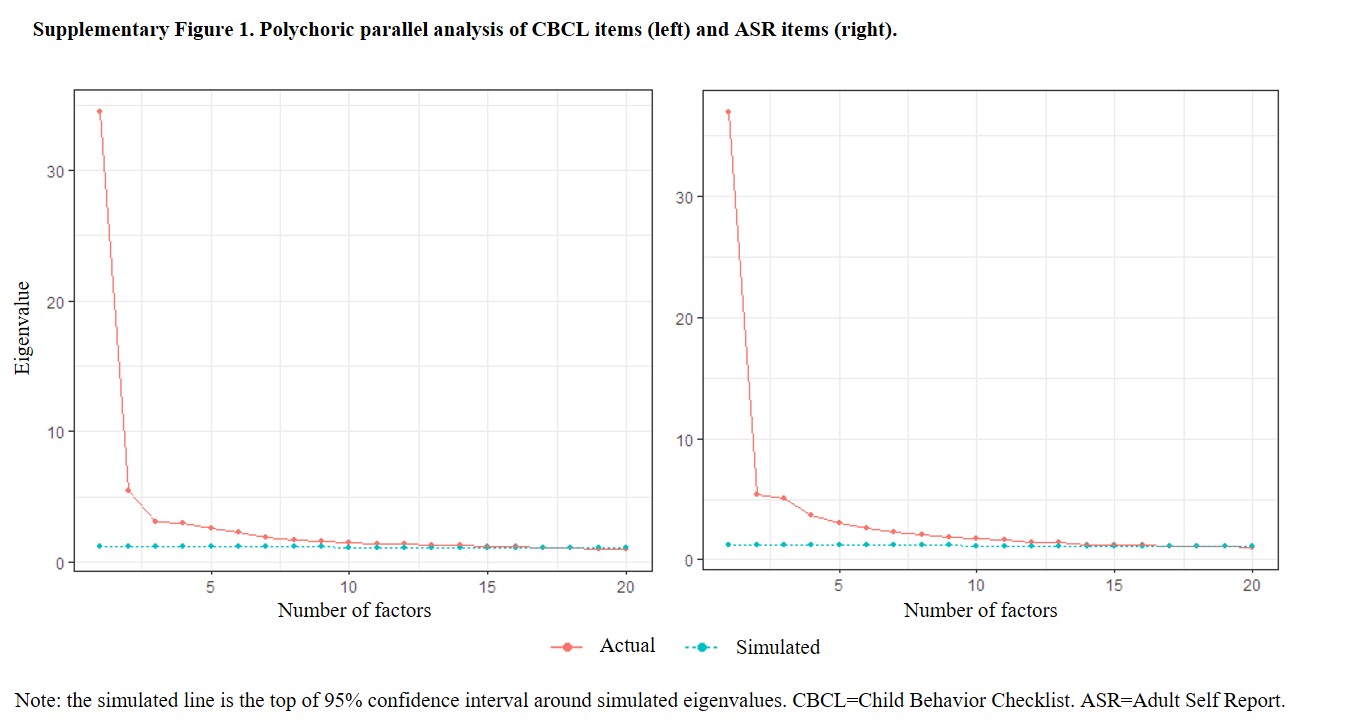

Supplement: Supplementary file 2 — Supplementary Figure 1 [file 41398_2019_593_MOESM2_ESM.jpg]
